# Perioperative Transfusion and Mortality for Cardiovascular Surgery: A Cohort Study Based on Population in Republic of Korea

**DOI:** 10.3390/jcm13082328

**Published:** 2024-04-17

**Authors:** Tak-Kyu Oh, In-Ae Song

**Affiliations:** 1 Department of Anesthesiology and Pain Medicine, Seoul National University Bundang Hospital, Seongnam 13620, Republic of Korea; airohtak@hotmail.com; 2 Department of Anesthesiology and Pain Medicine, College of Medicine, Seoul National University, Seoul 08826, Republic of Korea

**Keywords:** blood transfusion, mortality, surgery, intensive care units

## Abstract

**Objective:**This study aimed to evaluate the rate of transfusion for cardiovascular surgeries between 2010 and 2019 in Republic of Korea and the association between blood transfusion and postoperative mortality. **Methods:** Data were extracted from the National Health Insurance Service database in Republic of Korea. This study includes adult patients who underwent cardiovascular surgery between 1 January 2010 and 31 December 2019. The endpoints were in-hospital mortality and the 1-year all-cause mortality. **Results:** The analysis included 62,794 cases, with transfusions used in 88.8% of cases. Multivariable logistic regression revealed that older age, comorbidities, hospital admission through the emergency room, aortic procedures (versus coronary artery bypass grafting), cardiopulmonary bypass, repeat procedures, and supportive therapies during the intensive care (extracorporeal membrane oxygenation and mechanical ventilation) were risk factors for blood transfusion. Female sex was associated with a lower risk of transfusion. Perioperative blood transfusion was associated with a 6.87-fold increased risk of in-hospital mortality (odds ratio [OR]: 6.87, 95% confidence interval [CI]: 3.95, 11.93; *p* < 0.001) and a 3.20-fold increased risk of 1-year all-cause mortality (OR: 3.35, 95% CI: 2.75, 3.93; *p* < 0.001). **Conclusions:** Blood transfusion is used at a high rate in cardiovascular surgeries, and it was associated with increases in the risk of in-hospital and 1-year all-cause mortality. However, these correlations should be viewed with caution as emergent phenomena rather than causative. Understanding factors associated with the need for blood transfusion can assist surgeons in predicting the outcomes of cardiovascular surgery and in tailoring procedures as needed to optimize outcomes.

## 1. Introduction

Cardiovascular surgery is associated with a substantial risk of perioperative bleeding that necessitates blood transfusion. Blood transfusion is needed in 43–54% of cases of coronary artery bypass surgery (CABG), 54–67% of cases of valve surgery, 80–88% of cases of combined CABG and valve surgery, and 61–90% of cases of aortic surgery [1,2,3]. The risks and benefits of transfusion during cardiac or aortic surgeries remain debatable [4]. Hemorrhagic shock caused by hemorrhage and perioperative anemia are dangerous complications associated with post-surgery morbidity and mortality [5]. Theoretically, red blood cell (RBC) transfusion is thought to increase oxygen delivery to tissues and prevent myocardial ischemia in cases of major bleeding or anemia during perioperative cardiovascular surgery [6]. The most recent guideline recommended that physicians may set a serum hemoglobin threshold of 7.5 g/dL in determining RBC transfusion for patients undergoing cardiac surgery [7]. However, whether blood transfusion enhances tissue oxygenation remains debatable [6]. Despite corrected Hb levels, Turgeman et al. [8] found significantly poorer oxygenation and tissue perfusion after RBC transfusion in a single-center, retrospective study.

Infections, immunological reactions, pulmonary, renal, cardiac, and neurological complications, transfusion-related acute lung injury, and mortality are all associated with transfusion [4]. Transfusion-related harm has been reported in numerous studies on cardiac surgery and aortic procedures [4]. Compared to cardiac surgery, aortic surgery is more closely associated with perioperative bleeding and blood transfusion; however, the effect of blood transfusion on the postoperative mortality rate has not been well examined [2]. Furthermore, controversy exists regarding whether the transfusion of fresh frozen plasma (FFP), platelets, and cryoprecipitate is harmful or beneficial during the perioperative period of cardiovascular surgery, with conflicting results reported in available trials [9].

Accordingly, this study aimed to determine the relationship between perioperative blood and blood product transfusions and mortality and the risk factors for transfusion in cardiovascular surgery.

## 2. Materials and Methods

### 2.1. Study Design and Ethical Statement

This study was conducted in accordance with the Strengthening the Reporting of Observational Studies in Epidemiology [10] and was retrospective, population-based, cohort research. The institutional review board at Seoul National University Bundang Hospital, located in Seong-Nam-si, Republic of Korea, authorized the study protocol (approval ID: X-2302-808-901). The use of their anonymized data was approved by the National Health Insurance Service (NHIS) of Republic of Korea (Approval ID: NHIS-2021-1-620). Consent that was informed was not required.

### 2.2. Data Source and Study Population

The National Health Insurance Service (NHIS) is the sole insurer in Republic of Korea. The NHIS database contains information regarding the disease diagnosis, prescription of drugs, and procedures performed. Reimbursement for medical services in Republic of Korea requires that physicians submit the diagnosis and surgical codes in the NHIS database. Therefore, there are no missing data. The International Classification of Diseases, 10th Revision (ICD-10) codes are used for disease diagnoses.

Adult patients who underwent cardiovascular surgery between 1 January 2010 and 31 December 2019 constituted the study population. Table S1 contains a list of the codes and names of the surgeries in this study. Surgeries were classified into five groups: CABG only, the valve only, CABG + valve, aortic procedures, and others.

### 2.3. Blood Transfusion

Patients were categorized into two groups depending on whether they underwent blood transfusions before cardiovascular surgery. Transfusions of packed red blood cells (pRBCs), platelets (PLTs), FFP, and cryoprecipitate were recorded in the NHIS database using the associated transfusion codes. Although guidelines for perioperative blood transfusions have been established in Korea [11], transfusions at individual hospitals were based on the judgment of anesthesiologists and surgeons. The group that received a blood transfusion at the time of the procedure was defined as the BT group, and the group that did not receive a blood transfusion was defined as the non-BT group.

### 2.4. Endpoints

The primary endpoints were in-hospital mortality and the 1-year all-cause mortality according to the blood transfusion status. The secondary endpoints were in-hospital mortality and mortality within one year following cardiovascular surgery stratified by the type of blood transfusion used: pRBC, FFP, PLT, or cryoprecipitates.

### 2.5. Covariates

The following demographic and socioeconomic data were obtained from the NHIS database. Demographic data included age at the time of surgery, sex, and occupational status. Depending on whether the address was in a big city or another location, it was designated as an urban or rural address. The NHIS uses household income data from the database to assess a person’s eligibility for medical aid programs. Patients were categorized into five groups for analysis based on their medical aid status and income quartiles. Hospitals were classified into general and tertiary categories. However, to receive social assistance benefits in Republic of Korea, individuals with impairments are required to register with the NHIS database. Based on the severity of their difficulties, patients with disabilities are categorized into six groups inside the database. We classified the disability severity into two categories for analysis: severe, or grades 1–3, and mild-to-moderate, or grades 4–6. Based on the supplied ICD-10 codes, the Charlson Comorbidity Index (CCI) was used to assess comorbidities, as indicated in Table S2. Further data were gathered for analysis, including the primary or repeat procedure, emergency room (ER) admission, cardiopulmonary bypass (CPB) use during surgery, and postoperative supportive therapies such as mechanical ventilation, continuous renal replacement therapy (CRRT), extracorporeal membrane oxygenation (ECMO), and continuous renal replacement therapy.

### 2.6. Statistical Analysis

While categorical variables are shown as numbers and percentages, continuous variables are shown as mean values and standard deviations. A *t*-test was used to compare clinical characteristics between BT and non-BT groups for continuous variables and a chi-square test for categorical variables. Multivariable logistic regression was used to determine the possible risk factors for blood transfusion in patients undergoing cardiovascular surgery using an adjusted model that considered all covariates. Additionally, we built a multivariable logistic regression model to investigate the relationship between in-hospital mortality and blood transfusions. To investigate the relationship between blood transfusion and the 1-year all-cause mortality, we built a multivariable logistic regression model for the 1-year all-cause mortality. The results are presented as odds ratios (OR) with 95% confidence intervals (CI), considering the impacts of multicollinearity in the models with a variance inflation factor criteria of less than 2.0. R software (version 4.0.3, R packages, R Project for Statistical Computing, Vienna, Austria) was used to perform analyses, and a *p*-value < 0.05 was considered statistically significant.

## 3. Results

### 3.1. Study Population

**Figure 1:**
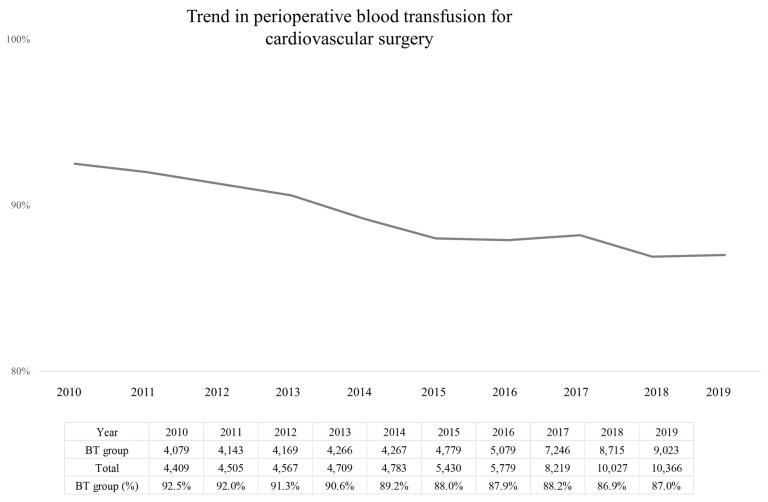
Trend in perioperative blood transfusion for cardiovascular surgery from 2010 to 2019.

The analysis included 62,794 surgical cases, contributed by 61,090 adult (≥18 years) patients, and there were no missing data. The trend in blood transfusion rate during cardiovascular surgery over the study period, from 2010 to 2019, is shown in Figure 1, with an overall decrease from 92.5% to 87% over the study period. The blood transfusion rate for each of the five surgical groups is shown in Figure S1. The blood transfusion rate was highest for the CABG + valve group (3046/3230, 94.3%), followed by the aortic procedure group (7331/7968, 92.0%), valve-only group (32,005/35,812, 89.4%), other group (4377/5080, 86.2%). The lowest transfusion rate was for the CABG-only group (9007/10,704, 84.1%).

The clinicopathological characteristics of patients are presented in Table 1. Overall, perioperative blood transfusion was required in 88.8% (55,766/62,794) of cases, forming the BT group, with cases not requiring blood transfusion forming the non-BT group. The proportion for each transfusion type was as follows: pRBC, 85.1% (53,453/62,794); FFP, 65.5% (41,157/62,794); PLT, 46.8% (29,377/62,794); and cryoprecipitate, 23.4% (14,704/62,794).

### 3.2. Factors Associated with Perioperative Blood Transfusion

The clinicopathological characteristics between the BT and non-BT groups are reported in Table 2, with outcomes of the multivariable logistic regression model for perioperative blood transfusion during cardiovascular surgery reported in Table 3.

### 3.3. In-Hospital Mortality and 1-Year All-Cause Mortality

Results of the survival analysis for in-hospital and 1-year all-cause mortality are reported in Table 4, Tables S3 and S4. In multivariable analysis (model 1), the BT group had a 6.87-fold higher risk of in-hospital mortality compared to the non-BT group (OR:6.87, 95% CI:3.95, 11.93; *p* < 0.001). In model 2, pRBC (OR:3.48, 95% CI:2.15, 5.65; *p* < 0.001), FFP (OR:2.06, 95% CI:1.80, 2.35; *p* < 0.001), PLT (OR:2.51, 95% CI:2.06, 3.07; *p* < 0.001), and cryoprecipitates (OR:1.58, 95% CI:1.42, 1.74; *p* < 0.001) transfusions were associated with an increased risk of in-hospital mortality. In model 3, the BT group had a 3.35-fold higher risk of 1-year all-cause mortality than the non-BT group (OR:3.35, 95% CI:2.75, 3.93; *p* < 0.001). In model 4, pRBC (OR:2.10, 95% CI:1.50, 2.51; *p* < 0.001), FFP (OR:1.58, 95% CI:1.44, 1.69; *p* < 0.001), PLT (OR:1.75, 95% CI:1.54, 1.88; *p* < 0.001), and cryoprecipitates (OR:1.34, 95% CI:1.27, 1.43; *p* < 0.001) transfusions were associated with an increased 1-year all-cause mortality.

## 4. Discussion

In this study, we identified a transfusion rate of 88.8% among 62,794 cases of cardiac or aortic surgery in Republic of Korea, which was greater than that reported in other countries [1,2]. After adjustment, transfusion was associated with increased in-hospital and 1-year all-cause mortality. An association was observed between higher mortality and the use of transfusion of each blood component (pRBC, FFP, platelets, and cryoprecipitate). However, these associations should be interpreted cautiously as emergent phenomena rather than causal. To our knowledge, this is the first study to examine the association between transfusion and mortality after cardiovascular surgery using big data from the NHIS in Republic of Korea. The factors associated with a greater risk for perioperative transfusion were older age, lower income, rurality, higher CCI, disabilities, admission through the ER, tertiary general hospital, aortic procedure (vs. CABG), CPB use during surgery, repeat surgeries, and highly equipped care in the ICU, such as mechanical ventilator care, ECMO support, and CRRT use. Notably, female sex was associated with a lower risk for transfusion.

Intraoperative pRBC transfusion rates in cardiovascular surgery patients ranged from 9% to 100% in 16 countries, while postoperative rates in 70 centers across these countries varied from 25% to 87% [12]. However, there has been a recent trend toward reducing perioperative transfusion rates associated with cardiovascular surgery through strict guidelines for blood transfusion [13]. Indeed, the rate of perioperative blood transfusions associated with cardiovascular surgery in the U.S. has slightly declined from 2011 to 2016 [14]. We are not sure why the blood transfusion rate associated with cardiovascular surgery is high in Korea, but it is possible that recent guidelines recommending restrictive transfusion practices have been slowly adopted in the Korean clinical setting.

Perioperative anemia is generally hazardous to patients. In non-cardiac surgery, perioperative anemia is associated with increased perioperative mortality in patients with cardiovascular illness compared to those without anemia [15]. In a retrospective analysis of patients who refused blood transfusions for religious reasons, mortality was low among patients with postoperative Hb levels ranging between 7.1 and 8.0 g/dL, with increasing mortality as Hb levels decreased. Mortality was particularly high when Hb levels fell below 5–6 g/dL [16]. In cardiac surgery, the lowest hematocrit level during CPB increases the risk for in-hospital mortality, as well as the risk of intra-aortic balloon pump placement and weaning failure from CPB after adjustment [17,18]. In their observational study, Defoe et al. [17] indicated that patients with a lower preoperative hematocrit were more likely to have a low hematocrit during CPB in CABG.

However, there are conflicting results regarding the harmful effects of anemia and blood transfusions on survival outcomes of cardiac and aortic surgeries. Spiess et al. [19] reported that a high hematocrit value ≥34% at ICU admission was associated with a higher rate of myocardial infarction, more severe left ventricular dysfunction, and mortality after adjustment for confounding factors. In their study, the rate of intraoperative transfusion using pRBC was similar between the high (≥34%) and low (<34%) hematocrit groups. However, the rate of postoperative transfusion of all blood products was higher for the low than high hematocrit group.

Perioperative anemia may be harmful to patients by reducing tissue oxygenation, consequently causing organ ischemia and failure. However, whether increasing the hematocrit from blood transfusions enhances tissue oxygenation is debatable [6,8]. Despite several investigations on transfusion strategies, namely liberal versus restricted, in cardiac surgery, hematocrit thresholds for transfusion have not yet been established. Notably, low Hb targets, such as 7–8 g/dL, have not been found to be inferior to high Hb targets [20].

In cardiac surgery, the transfusion of packed RBC has been associated with increased morbidity, prolonged hospital stay, and higher cost of hospitalization. Consequently, efforts have been made to establish blood preservation strategies [21,22,23,24]. Paone et al. [25] found that even a transfusion of only 1–2 units of RBC increases morbidity and mortality after off-pump CABG. Therefore, understanding the risk for major bleeding for different cardiac and aortic surgeries can assist surgeons in implementing appropriate blood preservation strategies.

Some pathophysiologic mechanisms have been suggested to explain the association between blood transfusion and increased mortality after cardiovascular surgery. First, transfusion-related acute lung injury (TRALI) may occur through white blood cell (WBC) antibodies targeting the recipient’s WBCs in the pulmonary microcirculation [26]. Although the exact incidence of TRALI is unknown, a prior study found that there is one case for every 100,000 units of product [27]. Second, within 24 h of a transfusion, an incompatible donor’s RBC may be destroyed intravascularly or extravascularly by a pre-existing circulating antibody, resulting in a hemolytic transfusion reaction [26]. The prevalence of hemolytic transfusion reactions is reported to be rare, and it contributed to 3.6% of total adverse transfusion reactions [28]. Third, fatal transfusion-acquired infection or sepsis may occur owing to transfusion [29]. While the risk of transfusion-transmitted infection is normally very low in industrialized countries (less than one in a million units), blood safety is still not guaranteed in developing countries, particularly in Africa [30].

In our study, the risk of major bleeding and transfusion was substantially higher for aortic surgery compared to cardiac surgery, consistent with the findings of previous studies [1,2,3]. However, transfusion-related research is more limited to cardiac than aortic surgeries. Blood preservation strategies have been developed to reduce the need for transfusion during aortic surgery [31,32]. In their study of 63 consecutive aortic replacement surgeries, Smith et al. [31] observed that a low preoperative hematocrit and endocarditis were predictors of mortality and that a low preoperative hematocrit and CPB duration were risk factors for transfusion. Chu et al. [32] further indicated that the use of a blood preservation strategy in surgeries to the ascending aorta and aortic arches reduced the need for blood transfusion and postoperative morbidity but not mortality. A systematic review and meta-analysis [33] provided quality evidence of the association of anemia with mortality in aortic surgeries. In a study on thoracoabdominal aneurysm repairs, Cambria et al. [34] identified that both postoperative renal injury and transfusion increased the risk of mortality. Hemli et al. [2] reported that 90% of patients who underwent root replacement for aortic dissection required blood transfusion and that transfusion was an independent predictor of increased mortality risk.

In addition to limited research on the association between transfusion and mortality in cardiac surgeries, little research has been conducted on the relationship between the use of different blood products for transfusion and mortality in cardiac and aortic surgeries. Most of the research in this regard has reported on the risks of morbidity associated with transfusion of platelets [9,35,36]. Although a systematic review did not identify an association between platelet transfusion and an increase in morbidity after cardiac surgery [35], Spiess et al. [19] and Fransen et al. [9] did report an association between platelet transfusion and an increased incidence of major perioperative adverse outcomes in patients undergoing CABG. In their study of patients undergoing valve surgery, Ming et al. [4] reported an increased risk of mortality for different transfusion components: FFP, platelets, and cryoprecipitate.

The limitations of our study need to be acknowledged when considering the implications for practice. Foremost is the retrospective design for which effects of selection bias cannot be denied, and causation cannot be attributed. Second, detailed information regarding the surgery and anesthesia, which can influence outcomes, is not provided in the NHIS database. Third, there might be unmeasured, unanalyzed, and potential confounders that were not included in the model. Fourth, generalizability could be limited, and our results may not be directly applicable to populations outside of Republic of Korea owing to differences in clinical practices, patient demographics, and healthcare systems. Lastly, we did not provide detailed information regarding the criteria for blood transfusions during the perioperative period, which could vary significantly between physicians and hospitals, potentially affecting the study’s outcomes.

## 5. Conclusions

In summary, the blood transfusion rate during cardiac and aortic surgeries in Republic of Korea is high at 88.8% and greater than that in other countries. Future studies will be needed to evaluate the reasons for this. Additionally, transfusion with RBC, FFP, platelets, and cryoprecipitate was associated with an increased risk of in-hospital and 1-year all-cause mortality. However, these associations should be interpreted cautiously as emergent phenomena rather than causal. Knowledge of factors associated with the need for blood transfusion can assist surgeons in predicting the outcomes of cardiovascular surgery and in tailoring procedures as needed to optimize outcomes. However, future research on this topic should be conducted in more diverse geographies and among populations with different demographic characteristics.

## Figures and Tables

**Table 1:** Clinicopathological characteristics of patients (62,794 cases for 61,090 patients).

Variable		Mean (SD) or Number (%)
Age, year		63.0 (13.8)
Sex, male		36,447 (58.0)
Having a job		33,599 (53.5)
Household income level		
	Medical aid program	3296 (5.2)
	Q1 (Lowest)	9678 (15.4)
	Q2	9346 (14.9)
	Q3	12,709 (20.2)
	Q4 (Highest)	19,833 (31.6)
	Unknown	7932 (12.6)
Residence		
	Urban area	24,706 (39.3)
	Rural area	31,224 (49.7)
	Unknown	6864 (10.9)
ICU stay, day		3.1 (3.6)
LOS, day		17.1 (9.3)
CCI, point		1.8 (1.7)
Underlying disability		
	Mild to moderate	5191 (8.3)
	Severe	4013 (6.4)
Hospital admission through ER		17,940 (28.6)
Result of hospitalization		
	Discharge and follow-up in same hospital	19,973 (31.8)
	Transfer to long-term facility care center	1052 (1.7)
	Death during hospitalization	2582 (4.1)
	Discharge and outpatient clinic follow-up	39,187 (62.4)
Type of hospital		
	Tertiary general hospital	48,922 (77.9)
	General hospital	13,872 (22.1)
Type of surgery		
	CABG only	10,704 (17.0)
	Valve only	35,812 (57.0)
	CABG + valve	3230 (5.1)
	Aortic procedures	7968 (12.7)
	Others	5080 (8.1)
CPB use during surgery		52,330 (83.3)
Redo case		1704 (2.7)
Mechanical ventilator support		59,850 (95.3)
ECMO support		2612 (4.2)
CRRT use		3737 (6.0)
1-year mortality		6393 (10.2)
Total cost for hospitalization, USD		24,964.6 (14,070.0)
Total transfusion		55,766 (88.8%)
	pRBC	53,453 (85.1%)
	FFP	41,157 (65.5%)
	Platelet transfusion	29,377 (46.8%)
	Cryoprecipitate transfusion	14,704 (23.4%)

SD, standard deviation; ICU, intensive care unit; LOS, length of hospital stays; CCI, Charlson comorbidity index; ER, emergency room; CABG, coronary artery bypass grafting; CPB, cardiopulmonary bypass; ECMO, extracorporeal membrane oxygenation; CRRT, continuous renal replacement therapy; USD, United States Dollars; pRBC, packed red blood cell; FFP, fresh frozen plasma.

**Table 2:** Comparison of clinicopathological characteristics between the BT and non-BT groups.

Variable		BT Group *n* = 55,766	Non-BT Group *n* = 7028	*p*-Value
Age, year		63.6 (13.7)	58.3 (13.5)	<0.001
Sex, male		31,112 (55.8)	5335 (75.9)	<0.001
Having a job		29,445 (52.8)	4154 (59.1)	<0.001
Household income level				<0.001
	Medical aid program	3060 (5.5)	236 (3.4)	
	Q1 (Lowest)	8601 (15.4)	1077 (15.3)	
	Q2	8209 (14.7)	1137 (16.2)	
	Q3	11,099 (19.9)	1610 (22.9)	
	Q4 (Highest)	17,388 (31.2)	2445 (34.8)	
	Unknown	7409 (13.3)	523 (7.4)	
Residence				<0.001
	Urban area	21,807 (39.1)	2899 (41.2)	
	Rural area	27,492 (49.3)	3732 (53.1)	
	Unknown	6467 (11.6)	397 (5.6)	
ICU stay, day		3.3 (3.7)	1.5 (1.1)	<0.001
LOS, day		17.7 (9.4)	11.8 (5.9)	<0.001
CCI, point		1.9 (1.7)	1.2 (1.4)	<0.001
Underlying disability				<0.001
	Mild to moderate	4736 (8.5)	455 (6.5)	
	Severe	3795 (6.8)	218 (3.1)	
Hospital admission through ER		16,997 (30.5)	943 (13.4)	<0.001
Result of hospitalization				<0.001
	Discharge and follow-up in same hospital	18,312 (32.8)	1661 (23.6)	
	Transfer to long-term facility care center	995 (1.8)	57 (0.8)	
	Death during hospitalization	2569 (4.6)	13 (0.2)	
	Discharge and outpatient clinic follow-up	33,890 (60.8)	5297 (75.4)	
Type of hospital				0.018
	Tertiary general hospital	43,369 (77.8)	5553 (79.0)	
	General hospital	12,397 (22.2)	1475 (21.0)	
CPB use during surgery		47,566 (85.3)	4764 (67.8)	<0.001
Redo case		1558 (2.8)	146 (2.1)	<0.001
Mechanical ventilator support		53,853 (96.6)	5997 (85.3)	<0.001
ECMO support		2600 (4.7)	12 (0.2)	<0.001
CRRT use		3721 (6.7)	16 (0.2)	<0.001
1-year mortality		6282 (11.3)	111 (1.6)	<0.001
Total cost for hospitalization, USD		25,928.8 (14,517.6)	17,313.7 (5525.4)	<0.001

ICU, intensive care unit; LOS, length of hospital stays; CCI, Charlson comorbidity index; ER, emergency room; CPB, cardiopulmonary bypass; ECMO, extracorporeal membrane oxygenation; CRRT, continuous renal replacement therapy; USD, United States Dollars

**Table 3:** Multivariable logistic regression model for perioperative blood transfusion during cardiovascular surgery.

Variable		OR (95% CI)	*p*-Value
Age, year		1.03 (1.03, 1.03)	<0.001
Sex, male		0.41 (0.39, 0.44)	<0.001
Having a job		0.99 (0.93, 1.05)	0.703
Household income level			
	Medical aid program	1.16 (0.98, 1.36)	0.080
	Q1 (Lowest)	1	
	Q2	0.95 (0.86, 1.04)	0.256
	Q3	0.89 (0.81, 0.97)	0.007
	Q4 (Highest)	0.84 (0.78, 0.92)	<0.001
	Unknown	0.91 (0.74, 1.12)	0.369
Residence			
	Urban area	1	
	Rural area	0.97 (0.92, 1.03)	0.336
	Unknown	1.72 (1.36, 2.17)	<0.001
CCI, point		1.26 (1.23, 1.29)	<0.001
Underlying disability			
	Mild to moderate	1.29 (1.16, 1.44)	<0.001
	Severe	2.13 (1.83, 2.47)	<0.001
Hospital admission through ER		2.12 (1.83, 2.47)	<0.001
Type of hospital			
	Tertiary general hospital	1	
	General hospital	0.90 (0.84, 0.96)	<0.001
Type of surgery			
	CABG only	1	
	Valve only	0.63 (0.56, 0.71)	<0.001
	CABG + valve	1.13 (0.94, 1.37)	0.191
	Aortic procedures	2.26 (1.99, 2.56)	<0.001
	Others	0.54 (0.47, 0.61)	<0.001
CPB use during surgery		3.66 (3.28, 4.09)	<0.001
Redo case		1.21 (1.01, 1.45)	0.039
Mechanical ventilator support		4.21 (3.79, 4.67)	<0.001
ECMO support		14.47 (8.15, 25.70)	<0.001
CRRT use		1.26 (1.23, 1.29)	<0.001
Year of surgery			
	2010	1	
	2011	0.93 (0.79, 1.09)	0.371
	2012	0.83 (0.71, 0.98)	0.026
	2013	0.75 (0.64, 0.88)	<0.001
	2014	0.64 (0.55, 0.75)	<0.001
	2015	0.56 (0.48, 0.65)	<0.001
	2016	0.55 (0.48, 0.64)	<0.001
	2017	0.58 (0.46, 0.61)	<0.001
	2018	0.53 (0.46, 0.60)	<0.001
	2019	0.52 (0.46, 0.60)	<0.001

OR, odds ratio; CI, confidence interval; CCI, Charlson comorbidity index; ER, emergency room; CABG, coronary artery bypass grafting; ECMO, extracorporeal membrane oxygenation; CRRT, continuous renal replacement therapy.

**Table 4:** Multivariable logistic regression model for in-hospital mortality, and multivariable Cox regression model for 1-year all-cause mortality.

Variable		OR (95% CI)	*p*-Value
Multivariable model 1 (in-hospital mortality)			
	Transfusion group (vs no transfusion group)	6.87 (3.95, 11.93)	<0.001
Multivariable model 2 (in-hospital mortality)			
	pRBC (vs no pRBC transfusion group)	3.48 (2.15, 5.65)	<0.001
	FFP (vs no FFP transfusion group)	2.06 (1.80, 2.35)	<0.001
	PLT transfusion (vs no PLT transfusion group)	2.51 (2.06, 3.07)	<0.001
	CPP transfusion (vs no CPP transfusion group)	1.58 (1.42, 1.74)	<0.001
Multivariable model 3 (1-year all-cause mortality)			
	Transfusion group (vs no transfusion group)	3.35 (2.75, 3.93)	<0.001
Multivariable model 4 (1-year all-cause mortality)			
	pRBC (vs no pRBC transfusion group)	2.10 (1.50, 2.51)	<0.001
	FFP (vs no FFP transfusion group)	1.58 (1.44, 1.69)	<0.001
	PLT transfusion (vs no PLT transfusion group)	1.75 (1.54, 1.88)	<0.001
	CPP transfusion (vs no CPP transfusion group)	1.34 (1.27, 1.43)	<0.001

OR, odds ratio; CI, confidence interval; pRBC, packed red blood cell; FFP, fresh frozen plasma; PLT, platelet; CPP, cryoprecipitate.

## Data Availability

Data will be available upon reasonable request to the corresponding author.

## Supplementary Materials

The following supporting information can be downloaded at: https://www.mdpi.com/article/10.3390/jcm13082328/s1, Table S1. Names of the cardiovascular surgeries and procedural codes used for data extraction. Table S2. The ICD-10 codes are used for comorbidity calculation to compute the Charlson comorbidity index. Table S3. All ORs with 95% CIs of other covariates in multivariable model 1. Table S4. All HRs with 95% CIs of other covariates in multivariable model 3. Figure S1. The rate of blood transfusion for each of the five surgical groups.
