# Supplementary material for: Perioperative Transfusion and Mortality for Cardiovascular Surgery: A Cohort Study Based on Population in Republic of Korea
Source: J Clin Med. 2024 Apr 17;13(8):2328. doi: 10.3390/jcm13082328 (PMC11051365; doi:10.3390/jcm13082328)
Supplement: Supplementary file 1 [file jcm-13-02328-s001.zip › Table S4.pdf]

Table S4. All HRs with 95% CIs of other covariates in multivariable model 3

Variable	HR (95% CI)	*P*-value
Age, year	1.04 (1.04, 1.05)	<0.001
Sex, male	1.07 (1.02, 1.13)	0.008
Having a job	0.97 (0.92, 1.03)	0.383
Household income level		
Q1 (Lowest)	1	
Q2	0.95 (0.87, 1.05)	0.349
Q3	0.88 (0.81, 0.97)	0.007
Q4 (Highest)	0.90 (0.82, 0.97)	0.008
Medical aid program group	1.24 (1.10, 1.39)	<0.001
Unknown	0.89 (0.72, 1.10)	0.270
Residence		
Urban area	1	
Rural area	0.96 (0.91, 1.01)	0.136
Unknown	1.51 (1.22, 1.87)	<0.001
CCI, point	1.09 (1.08, 1.11)	<0.001
Hospital admission through ER	1.64 (1.56, 1.73)	<0.001
Type of hospital		
Tertiary general hospital	1	
General hospital	1.17 (1.10, 1.24)	<0.001
Type of surgery		
CABG only	1	
Valve only	1.30 (1.19, 1.42)	<0.001
CABG + valve	1.30 (1.14, 1.47)	<0.001
Aortic procedures	1.96 (1.79, 2.15)	<0.001

Others	1.19 (1.06, 1.33)	0.003
CPB use during surgery	0.91 (0.83, 0.99)	0.028
Redo case	0.86 (0.73, 1.02)	0.082
Mechanical ventilator support	1.07 (0.92, 1.24)	0.383
ECMO support	6.70 (6.27, 7.15)	<0.001
CRRT use	4.33 (4.07, 4.61)	<0.001
Year of admission		
2010	1	
2011	1.12 (0.98, 1.28)	0.110
2012	1.07 (0.93, 1.23)	0.338
2013	0.93 (0.81, 1.07)	0.291
2014	1.05 (0.92, 1.20)	0.489
2015	0.96 (0.84, 1.10)	0.554
2016	0.80 (0.70, 0.91)	<0.001
2017	0.78 (0.69, 0.88)	<0.001
2018	0.75 (0.67, 0.85)	<0.001
2019	0.75 (0.67, 0.85)	<0.001

HR, hazard ratio; CI, confidence interval; CCI, Charlson comorbidity index; ER, emergency room; CABG, coronary artery bypass grafting; CPB, cardiopulmonary bypss; ECMO, extracorporeal membrane oxygenation; CRRT, continuous renal replacement therapy; pRBC, packed red blood cell; FFP, fresh frozen plasma.
