# Supplementary material for: Perioperative Transfusion and Mortality for Cardiovascular Surgery: A Cohort Study Based on Population in Republic of Korea
Source: J Clin Med. 2024 Apr 17;13(8):2328. doi: 10.3390/jcm13082328 (PMC11051365; doi:10.3390/jcm13082328)
Supplement: Supplementary file 1 [file jcm-13-02328-s001.zip › TableS1.pdf]

Table S1. Names of the cardiovascular surgeries and procedural codes used for data extraction

O1793	Valve Replacement-Aortic Valve
O1782	Valvuloplasty-Mitral Valve
O1792	Valve Replacement-Mitral Valve
O2031	Resection of Aneurysm-Ascending Aorta
O1781	Valvuloplasty-Tricuspid Valve
OA641	Vascular Bypass Operation (Aorta-Coronary), Simple
OA649	Vascular Bypass Operation (Aorta-Coronary)-Simple (Off Pump CABG)
OA648	Vascular Bypass Operation (Aorta-Coronary)-Simple (Off Pump CABG)
O1981	Resection of Atrial Myxoma
O2034	Resection of Aneurysm-Abdominal Aorta and Iliac Artery
O2032	Resection of Aneurysm-Aortic Arch
O1648	Vascular Bypass Operation (Aorta-Coronary)-Simple
O2033	Resection of Aneurysm-Descending Thoracic Aorta
O1795	Reoperation of Valvuloplasty-Mitral Valve
O1641	Vascular Bypass Operation (Aorta-Coronary), Simple
OA640	Vascular Bypass Operation (Aorta-Coronary)-Simple (Off Pump CABG)
O1640	Vascular Bypass Operation (Aorta-Coronary)-Simple
O1644	Vascular Bypass Op (Aorta-Renal, Thoracic, Abdominal Aorta-Femoral, Aorta-Splanchnic),Artificial Vessel
O1796	Reoperation of Valvuloplasty-Aortic Valve
Q8080	Heart Transplantation
O1799	Sutureless Aortic Valve Replacement
O1649	Vascular Bypass Operation (Aorta-Coronary)-Simple
O0224	Resection of Aneurysm-Abdominal Aorta (Infrarenal)
O1982	Resection of Cardiac Tumor-Others
O1797	Valve Replacement-Pulmonary Valve
O1783	Valvuloplasty-Aortic Valve

O1791	Valve Replacement-Tricuspid Valve
O1798	Reoperation of Valvuloplasty-Pulmonary Valve
O1794	Reoperation of Valvuloplasty-Tricuspid Valve
O1647	Vascular Bypass Operation (Aorta-Coronary), Complex
OA647	Vascular Bypass Operation (Aorta-Coronary), Complex
O0223	Resection of Aneurysm-Abdominal Aorta (Suprarenal [Juxtarenal])
O1830	Coronary Endarterectomy
OB638	Angioplasty (With Patch Graft)-Others, Artificial Vessel
OB634	Angioplasty (With Patch Graft)-By Thoracotomy, Artificial Vessel
O1680	Correction of Coarctation of Aorta
O1643	Vascular Bypass Op (Aorta-Renal, Thoracic, Abdominal Aorta-Femoral,Aorta-Splanchnic),Autologous Vessel
O1740	Operation of Aortic Stenosis
OB633	Angioplasty (End-to-End Anastomosis)-Others
OB639	Angioplasty (With Patch Graft)-Others, Autologous Vessel
OB631	Angioplasty (End-to-End Anastomosis)-By Thoracotomy
O0174	Vascular Bypass Operation (Aorta-innominate, carotid and subclavian arteries)
OB632	Angioplasty (End-to-End Anastomosis)-By Laparotomy
O0173	Vascular Bypass Operation (Aorto to carotid and subclavian artery)
O1852	Repair of Complicated Congenital Heart Diseases-Others
OB636	Angioplasty (With Patch Graft)-By Laparotomy, Artificial Vessel
Q8103	Lung Transplantation-Heart and Lung
OB635	Angioplasty (With Patch Graft)-By Thoracotomy, Autologous Vessel
OB637	Angioplasty (With Patch Graft)-By Laparotomy, Autologous Vessel
O1851	Repair of Complicated Congenital Heart Diseases-Highly Complicated
O1960	Closure of Aorto-Pulmonary Window
