# Supplementary material for: Perioperative Transfusion and Mortality for Cardiovascular Surgery: A Cohort Study Based on Population in Republic of Korea
Source: J Clin Med. 2024 Apr 17;13(8):2328. doi: 10.3390/jcm13082328 (PMC11051365; doi:10.3390/jcm13082328)
Supplement: Supplementary file 1 [file jcm-13-02328-s001.zip › Table S3.pdf]

Table S3. All ORs with 95% CIs of other covariates in multivariable model 1

Variable	OR (95% CI)	*P*-value
Age, year	1.04 (1.04, 1.05)	<0.001
Sex, male	0.98 (0.89, 1.08)	0.726
Having a job	0.97 (0.86, 1.08)	0.530
Household income level		
Q1 (Lowest)	1	
Q2	1.05 (0.88, 1.26)	0.567
Q3	0.99 (0.84, 1.17)	0.872
Q4 (Highest)	0.96 (0.83, 1.12)	0.623
Medical aid program group	1.37 (1.09, 1.70)	0.006
Unknown	1.01 (0.69, 1.49)	0.949
Residence		
Urban area	1	
Rural area	0.87 (0.78, 0.96)	0.006
Unknown	0.63 (0.42, 0.94)	0.024
CCI, point	1.04 (1.01, 1.07)	0.008
Hospital admission through ER	2.07 (1.88, 2.28)	<0.001
Type of hospital		
Tertiary general hospital	1	
General hospital	1.40 (1.25, 1.56)	<0.001
Type of surgery		
CABG only	1	
Valve only	1.31 (1.10, 1.56)	0.002
CABG + valve	1.47 (1.16, 1.87)	0.002
Aortic procedures	2.77 (2.33, 3.28)	<0.001

Others	0.86 (0.68, 1.08)	0.198
CPB use during surgery	1.09 (0.93, 1.28)	0.287
Redo case	1.45 (1.16, 1.88)	<0.001
Mechanical ventilator support	0.88 (0.67, 1.15)	0.343
ECMO support	23.08 (20.58, 25.89)	<0.001
CRRT use	7.99 (7.16, 8.93)	<0.001
Year of admission		
2010	1	
2011	1.04 (0.82, 1.31)	0.772
2012	0.88 (0.70, 1.12)	0.299
2013	0.67 (0.53, 0.86)	0.001
2014	0.77 (0.61, 0.98)	0.032
2015	0.73 (0.58, 0.92)	0.008
2016	0.61 (0.48, 0.77)	<0.001
2017	0.48 (0.38, 0.60)	<0.001
2018	0.47 (0.38, 0.58)	<0.001
2019	0.47 (0.38, 0.58)	<0.001

OR, odds ratio; CI, confidence interval; CCI, Charlson comorbidity index; ER, emergency room; CABG, coronary artery bypass grafting; CPB, cardiopulmonary bypss; ECMO, extracorporeal membrane oxygenation; CRRT, continuous renal replacement therapy; pRBC, packed red blood cell; FFP, fresh frozen plasma.
